# The Global Vaccine Safety Initiative: enhancing vaccine pharmacovigilance capacity at country level

**DOI:** 10.2471/BLT.14.138875

**Published:** 2014-07-31

**Authors:** Christine G Maure, Alexander N Dodoo, Jan Bonhoeffer, Patrick LF Zuber

**Affiliations:** a Department of Essential Medicines and Health Products, World Health Organization, Avenue Appia 20, 1211 Geneva 27, Switzerland.; b Centre for Tropical Clinical Pharmacology and Therapeutics, University of Ghana, Accra, Ghana.; c Brighton Collaboration Foundation, Basel, Switzerland.

## Vaccine safety

Large-scale immunization programmes now extend to all parts of the world, including least developed countries, where they reach unprecedented numbers of people. The number of vaccine doses administered worldwide continues to increase as new vaccines are developed and made available, and more people have access to immunization services. Development of immunization programmes in low- and middle-income countries (LMICs) has increased immunization coverage, which in turn has led to a reduction in vaccine-preventable diseases. However, as vaccine use has increased in LMICs, so has public attention to vaccine safety issues, as happened previously in high-income countries. This has created additional vulnerability for all immunization programmes. Whether or not they are well founded, concerns about serious adverse events following immunization may rapidly undermine public confidence and become a serious threat to effective vaccination strategies, eroding the enormous gains in disease control achieved with decades of effort.

Patterns of global access to vaccines and programme implementation are evolving. In the past, vaccines that became available for programmes in LMICs had already been used for years in high-income countries. Currently, however, some newly developed vaccines (e.g. rotavirus vaccine) have become available in LMICs only a short time after being introduced in high-income countries. Other new vaccines, such as meningococcal A vaccine or candidate vaccines against dengue and malaria, may be introduced first in LMICs, where the capacity for adequate safety monitoring is limited. Some of these vaccines may contain elements that have not been widely used in humans, including multiple antigens or recombinant viral vectors. As a result, for the first time people in LMICs are being administered with newly developed vaccines in settings where monitoring capacity is limited. In addition, there are important population groups for which the safety profile of vaccines may differ; for example, people with immune deficiency (in areas with high rates of human immunodeficiency virus prevalence), malnutrition and chronic conditions, as well as pregnant women. Although toxicity and safety are monitored in pre-licensing studies, the number of vaccine recipients observed in such studies is limited. Also, because serious vaccine reactions are very rare, it is generally only after licensure and administration to millions of people that such reactions are detected. Thus, a well-designed and well-structured global approach that involves adequate monitoring of large populations is required for early recognition of potential issues, timely investigation of concerns, informed decision-making and corrective actions.

## Optimizing vaccine safety

The Decade of Vaccines, which was launched in 2010, aims to increase coordination within the vaccine community worldwide. The Global Vaccine Action Plan^1^ – the framework endorsed by the World Health Assembly for the Decade of Vaccines – includes a vaccine safety strategy, the Global Vaccine Safety Blueprint.^2^

The aim of the blueprint is to enhance the safety of vaccines through effective use of pharmacovigilance principles and methods. Its three strategic goals are: to assist LMICs to have at least minimal capacity for vaccine safety activities; to enhance capacity for vaccine safety assessment in countries that introduce newly developed vaccines, that introduce vaccines in settings with novel characteristics, or that manufacture and use prequalified vaccines; and to establish a global support structure for vaccine safety. The blueprint proposes eight complementary strategic objectives. Four of these objectives aim to improve the technical aspects of spontaneous reporting, active surveillance and risk communication; and to ensure the availability of harmonized methods and tools. The remaining four objectives promote the establishment of effective managerial principles to facilitate international collaboration and information exchange relating to vaccine safety monitoring. Implementing the blueprint is a task that requires coordinated participation of vaccine safety stakeholders worldwide. To that end, the World Health Organization (WHO) launched the Global Vaccine Safety Initiative in March 2012.

In its initial phase, the Global Vaccine Safety Initiative is attempting to build a global support structure by linking existing vaccine safety initiatives. Numerous projects are already addressing one or more of the blueprint’s strategic objectives. Some of the top priorities for the initiative are to identify such projects and engage their sponsors in collaboration, and to help disseminate their products and experiences. Therefore, a Global Vaccine Safety Initiative portfolio of activities has been assembled, where activities are prioritized based on their expected impact, geographical relevance, feasibility, usefulness and sustainability.^3^ For each activity, the portfolio recognizes the roles of initiators, managers and donors. All stakeholders in global pharmacovigilance can use this portfolio to help identify ongoing efforts, allow for better synergies, minimize duplications and enable resource mobilization.

## A cooperative approach

Capacity development at country level is the main guiding principle of the Global Vaccine Safety Initiative. The initiative is aligned with other related WHO capacity-building efforts, including the strengthening of immunization programmes and national regulatory authorities. The WHO global vaccine safety resource centre is a website^4^ that provides on-site and distance-learning opportunities to public health officials, immunization programme managers, vaccination staff and members of committees reviewing adverse events following immunization. To provide countries with coordinated support, a roster of vaccine pharmacovigilance experts with appropriate cultural awareness and geographical location is being developed.

A network of countries from all six WHO regions has been created to share experiences in improving the detection and investigation of adverse events following immunization. These countries, together with the WHO Collaborating Centre for International Drug Monitoring (Uppsala Monitoring Center), have recently provided the rationale for a harmonized set of core variables that have been endorsed by WHO’s Global Advisory Committee on Vaccine Safety, for the surveillance of adverse events following immunization.^5^ One main benefit of this proposed reporting format is to encourage standardized reporting, in preparation for a harmonized web-based interface. This will allow countries to maintain their own database on adverse events following immunization while exchanging relevant information internationally.

For the purpose of analysing adverse events following immunization, the Brighton Collaboration case definitions^6^ are being piloted for use in LMIC settings. New definitions are also being developed to adapt to the conditions of interest for different vaccines. The Brighton Collaboration network provides a useful platform for developing and evaluating new approaches and tools, monitoring public confidence and conducting international studies investigating vaccine safety concerns. Building on the model of the pharmacovigilance toolkit – a web-based repository of resources and information – the WHO Collaborating Centre for Advocacy and Training in Pharmacovigilance in Accra, Ghana, has developed a similar toolkit that is useful in vaccine safety monitoring.^7^

The engagement of vaccine manufacturers in informing the development of harmonized tools is critical for optimizing the usefulness of those tools and their adoption by the private sector. Therefore, a Working Group on Vaccine Safety was established in 2013 with the Council for International Organizations of Medical Science. This Working Group provides a forum for information exchange between industry and other stakeholders within the Global Vaccine Safety Initiative; it also acts as a forum to devise approaches for transparent and active public–private interaction.

Increasing the availability of, and access to, computerized medical records would enhance vaccine safety. Various studies piloting different aspects of international collaboration in sharing such records have underpinned the feasibility and potential of such collaboration. There is a need to develop shared criteria for structural and procedural system requirements for international data access and exchange, including current technical, ethical, governance and scientific standards. Several studies are exploring the feasibility of using a shared infrastructure to establish the background rate of medical events of interest and allow for hypothesis testing in LMICs. A Global Vaccine Safety Initiative multi-country collaborative study is currently exploring the usefulness of a hospital-based network for case-only study designs.

Innovative approaches are needed to monitor public confidence in vaccination programmes,^8^ to permit professionals to predict and potentially initiate an intervention for local concerns about such programmes. Models to monitor public perception are being tested as a complementary method to passive surveillance. The understanding of the origins, drivers and dynamics of public concerns, and their impact on coverage, could both inform public health responses, and provide insights and guidance for future vaccine introduction programmes.

The Decade of Vaccines provides a unique opportunity for moving the global vaccine safety agenda forward. The increasing interest of many stakeholders engaged with immunization programmes in LMICs provides visibility for the blueprint strategies. Efforts aimed at building vaccine safety capacity could ultimately be leveraged for the development of systemic and sustainable pharmacovigilance that should benefit all health products. As a result, the world will be closer to a more equitable way of ensuring the safety of health products, wherever in the world they are administered.
